# Biological Evaluation and Structural Analysis of Some Aminodiphenylamine Derivatives

**DOI:** 10.3390/antiox12030713

**Published:** 2023-03-13

**Authors:** Alexandru Bujor, Anamaria Hanganu, Victorita Tecuceanu, Augustin M. Madalan, Madalina Tudose, Luminita Marutescu, Marcela Popa, Carmen Mariana Chifiriuc, Irina Zarafu, Petre Ionita

**Affiliations:** 1 Faculty of Chemistry, University of Bucharest, 90 Panduri, 050663 Bucharest, Romania; alexandru.bujor@drd.unibuc.ro (A.B.); anamaria.hanganu@unibuc.ro (A.H.); augustin.madalan@chimie.unibuc.ro (A.M.M.); irina.zarafu@chimie.unibuc.ro (I.Z.); 2 ‘C. D. Nenitzescu’ Institute of Organic and Supramolecular Chemistry of the Romanian Academy, 202B Spl. Independentei, 060023 Bucharest, Romania; vtecu@ccocdn.ro; 3 Institute of Physical Chemistry, 202 Spl. Independentei, 060021 Bucharest, Romania; 4 Faculty of Biology, University of Bucharest, 91-95 Spl. Independentei, 050095 Bucharest, Romania; luminita.marutescu@bio.unibuc.ro (L.M.); marcela.popa@bio.unibuc.ro (M.P.); carmen.chifiriuc@bio.unibuc.ro (C.M.C.)

**Keywords:** diphenylamine, antioxidant, lipophilicity, NBD, hydrophobicity, RP-TLC

## Abstract

4-Aminodiphenylamino derivatives were investigated for their antioxidant and hydrophobicity character, together with other biological measurements, such as antimicrobial and antibiofilm activity. Among these nine compounds used, we obtained novel derivatives via reaction of the starting material with NBD-chloride. Therefore, we performed a full structural analysis for these compounds, i.e., elemental analysis, IR, UV-Vis, ^1^H- and ^13^C-NMR, ESI-MS, X-ray diffraction on single crystal, etc. The hydrophobicity of all the compounds was measured either experimentally using the RP-TLC technique, or via calculation using the fragments method. The other structural characteristics were analyzed, and a correlation between the experimental and computed properties was found. Moreover, the results of the biological evaluation showed that some of the synthesized compounds have antimicrobial and antibiofilm activity.

## 1. Introduction

Diphenylamine derivatives are industrial compounds with many applications, such as antioxidants, dye starting materials, medicine precursors, and so on. Therefore, they are easily accessible and synthesized in huge quantities [1,2,3,4,5]. The structure of such derivatives plays an important role in their actual properties, and a correlation between their structure and activity exists. This correlation plays an important role in the study of biological interaction [6]. The total antioxidant capacity (*TAC*) and lipophilicity are among some of the most important molecular properties, which in quantitative structure–activity relationships (QSAR) are strongly correlated with biological activity. Lipophilicity (also well-known as hydrophobicity) is linked with drug absorption capacity, toxicity, metabolism, and other drug-receptor interactions [7].

*TAC* measurements are related to the capacity to fight reactive free radicals or other oxidant non-radical species [8]. For example, in the literature data, the term reactive oxygen species (ROS) refers to a wide range of chemicals, such as hydroxy radicals, hydroperoxides, peroxides, singlet oxygen, and so on [9]. All of these are highly reactive, and scholars are constantly searching for compounds that can reduce or cancel their action. In biological systems, antioxidants, such as ascorbic acid (vitamin C), polyphenols, or other amine derivatives, are known for their beneficial role in defending and sustaining cells viability [10].

One can perform a *TAC* analysis using several methods, such as the well-known DPPH (2,2-diphenyl-1-picrylhydrazyl free radical) method or ABTS (2,2′-azinobis-(3-ethylbenzothiazoline-6-sulfonic acid); the other methods are Folin-Ciocalteu or the ferric reducing antioxidant power (FRAP) test. All these analytical methods might have different mechanisms and can be based on different chemical reactions following different kinetics or mechanisms. Their advantages are their spectrophotometric measurements, meaning that specific wavelength adsorption monitors the processes. Moreover, these assays are applied when determining the antioxidant capacity of complex samples. 

Scholars must know the biological evaluation and measurements to know the lipophilicity (or hydrophobicity) of the compounds, which is dependent on their capacity to cross the lipophile cell membrane. Experimentally, scholars can achieve this by using the classical partition method, placing compounds between an immiscible polar and non-polar solvent pair (usually *n*-octanol-water) [11], or by using reversed-phase thin layer chromatography (RP-TLC), which is often employed to evaluate the organic water partitioning properties of solutes [12]. However, one can evaluate these parameters by using computer predictors and programs based on computational calculus. This approach is the fastest, but sometimes the predictions are quite different from the experimental values. In addition to lipophilicities, other computational parameters are easily accessible with a range of computing methods, including empirical ones [13].

**Figure 1:**
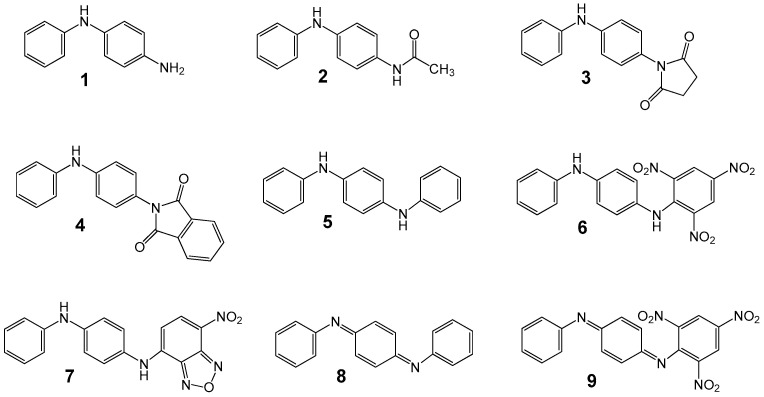
Chemical structures of the compounds used in this study.

Based on this information, we employed nine compounds (derived from aminodiphenylamine **1**, Figure 1) to evaluate their antioxidant capacity and lipophilicity, and to correlate this with some of their structural characteristics, as such, the derivatives contained different substituent groups, such as amide, nitro, and furazan heterocycle. Additionally, we used other biological activity tests, such as MIC (minimum inhibitory concentrations), MBC (minimum bacterial concentrations), and antibiofilm activity, against four bacterial reference stains.

## 2. Materials and Methods

### 2.1. Materials, Apparatus and Techniques Used

All reagents, solvents, and chemical materials were purchased from Merck and Chimopar and used as received. Compounds **1** and **5** were commercially available; compounds **2**–**4**, and **8** are known in the literature and we therefore synthesized them following similar methods [14,15,16,17]; compounds **7** and **9** are new derivatives obtained, and their physico-chemical characteristics are presented in detail. The purity of all compounds was checked with TLC (single spot).

UV-Vis measurements were performed in methanol, using a UVD-3500 UV-Vis double-bean spectrophotometer. 

IR spectra were measured using a Bruker Tensor 27 FT-IR spectrometer.

NMR spectra were measured in chloroform-*d*_1_ or DMSO-*d*_6_ using a Bruker Advance spectrometer operating at 500 MHz for ^1^H and 125 MHz for ^13^C. We report the chemical shifts *δ* as ppm values and the residual solvent peaks were used as an internal reference. For the MS spectra, we used a Varian 310—MS LC/MS/MS triple quadrupole mass spectrometer fitted with an electrospray ionization interface (ESI).

#### X-ray Crystallographic Analysis

X-ray diffraction measurements of compound **7** were performed with a Rigaku XtaLAB Synergy-S diffractometer operating with Mo-Kα (λ = 0.71073 Å) micro-focus sealed X-ray tube. We determined the structure by direct methods and refined it by using a full-matrix least squares technique based on *F*^2^. The non-H atoms were refined with anisotropic displacement parameters. Calculations were performed using the SHELX-2018 crystallographic software package. Table 1 provides a summary of the crystallographic data and the structure refinement for compound **7**. CCDC reference number: 2209847.

### 2.2. Synthesis of the Compounds ***7*** and ***9***

As mentioned before, compounds **1** and **5** are commercially available, while compounds **2**–**4**, **6**, and **8** were synthesized following the literature data [14,15,16,17]. Therefore, for these, the synthetic details and structural analysis are shown in the Supplementary Materials.

*Compound* **7**, *N^1^-(7-nitrobenzo[c][1,2,5]oxadiazol-4-yl)-N^4^-phenylbenzo-1,4-diamine*. 0.2 g (1 mmol) of NBD-chloride (4-chloro-7-nitrobenzofurazan) was added to 0.2 g (1.1 mmol) of 1 in 50 mL acetonitrile, followed by 0.5 g of sodium hydrogen carbonate. After mixing for 24 h, 200 mL of DCM was added together with 200 mL of water. Extraction was performed, and the organic phase was separated, dried over anhydrous sodium sulfate, filtered off, and the solvent was removed. Separation on silica-gel column using DCM as eluent afforded the pure compounds as crystals. Yield 46%. Melting point: 178 °C. ^1^H-NMR (500 MHz, CDCl_3_ + DMSO-*d*_6_, *δ* ppm, *J* Hz): 10.64 (s, 1H, N*H*), 8.35 (dd, 1H, H_Ar_, *J* = 1.5 Hz, *J* = 8.9 Hz), 7.78 (s, 1H, N*H*), 7.22–7.16 (m, 4H, H_Ar_), 7.11–7.06 (m, 4H, H_Ar_), 8.81 (t, 1H, H_Ar_, *J* = 6.8 Hz), 6.53 (dd, 1H, H_Ar_, *J* = 1.5 Hz, *J* = 8.9 Hz) ppm. ^13^C-NMR (125 MHz, CDCl_3_ + DMSO-*d*_6_, *δ* ppm): 144.37, 143.85, 142.66, 142.39, 142.31, 136.42, 128.67, 128.45, 124.75, 122.05, 120.22, 117.42, 116.53, 99.98. Elemental analysis for C_18_H_13_N_5_O_3_ Mass 347: calculated: C62.24%; H3.77%; N20.16%; found C62.61%; H3.75%; N19.87%. -ESI-MS: 346 (M-H^+^).

*Compound* **9**, *phenyl-picryl-p-phenylenediimine.* 0.3 g (0.75 mmol) of 6 dissolved in 60 mL DCM was stirred for 1 h with 10 g of lead dioxide. Filtration and removal of the solvent afforded the desired compound. Yield 85%. ^1^H-NMR (300 MHz, DMSO-*d_6_*, *δ* ppm, *J* Hz): 9.18 (s, 2H, H_Ar_), 7.46 (t, 2H, H_Ar_, *J* = 7.7 Hz), 7.27 (d, 2H, H_Ar_, *J* = 8.0 Hz), 7.06–6.95 (m, 5H, H_Ar_) ppm. ^13^C-NMR (75 MHz, DMSO-*d*_6_, *δ* ppm): 160.31, 156.83, 149.37, 143.03, 141.93, 139.93, 130.13, 128.23, 124.96, 124.84, 121.50, 119.56.

### 2.3. Experimental Lipophilicity Measurements

Briefly, 10 cm× 10 cm RP-TLC plates (C18, F254) were spotted with compounds **1**–**9** dissolved in methanol and eluted with a mixture of acetone-water (6/4, *v*/*v*) in a chromatographic tank. We repeated the procedure for different mixtures of acetone-water (7/3, 8/2, 9/1, *v*/*v*). Acetone was chosen over methanol due to the better solubility of the compounds, as well as better migration observed for all compounds **1**–**9**. The front elution was in all cases approximately 8 cm. The *R_f_* values were measured either by direct observation of the spots (for the colored compounds) or by using an UV-lamp. These values were converted into *R_M_* values using Equation (1), and we obtained the *R*_*M*0_ and *b* values by linear regression using Equation (2).

The lipophilicity and partition measurements were performed in two ways, using computational programs for the *log p* values and the experimental procedure of RP-TLC. For lipophilicity index *R_M_*_0_, we employed these measurements because of their simplicity and rapidity. For RP-TLC, we used a non-polar stationary phase, C18 derivatized silica-gel, and as eluent, we used a mixture of two solvents, acetone/water. Scholars widely use such settings to measure the experimental lipophilicity (*R_M_*_0_) and specific hydrophobic surface (*b*) using Equations (1) and (2).

(1) $$R_{M}=\log(\frac{1}{R_{F}}-1)$$

(2) $$R_{M}=R_{M_{0}}+bC$$

### 2.4. Total Antioxidant Capacity (TAC) Measurements

Stock solutions of compounds **1**–**9** were obtained in methanol at a concentration of 1 mg/mL. We also obtained a stock solution of 2,2-diphenyl-1-picrylhydrazyl free radical (DPPH) in the same solvent, at a concentration of 2 × 10^−4^ M. To 1.8 mL of DPPH solution was added 0.2 mL of each solution of the compounds **1**–**9** and we left the mixture in the dark for 30 min, followed by measurements of the absorbance at λ_max_ 517 nm. We obtained the *TAC* values using Equation (3), which is a common way to assess the antioxidant activity [18,19].

A second TAC evaluation was conducted using the ABTS assay, in which DPPH free radical was replaced by ABTS radical cation. While the stock solutions of the compounds **1**–**9** were the same, the ABTS radical cation was obtained by mixing (in a volumetric ratio one to one) a 7 mM aqueous solution of the ABTS parent compound and a 2.45 mM aqueous solution of potassium persulfate, and then allowing the reaction to settle overnight [20], to generate the ABTS radical cation.

### 2.5. Computational Measurements

To evaluate some properties that are easily accessible *via* computational studies, we used the package ChemBio3D Pro from Cambridge soft [21] (version 12.0.2) in the first instance. Each molecular structure was first minimized the energy using the MM2 job, and then the atomic charges were computed using the extended Huckel method. Correspondingly, the *log P* and *PSA* (polar surface area) values could be calculated using the online software MolInspiration [22].

### 2.6. Determination of MICs and MBCs of the Compounds

The broth microdilution method as described by the Clinical and Laboratory Standards Institute (CLSI, 2022 [23]) was used for the quantitative measurement of the antimicrobial activity of the tested compounds against four bacterial reference strains: *Staphylococcus aureus* ATCC 25923, *Enterococcus faecalis* ATCC 29212, *Escherichia coli* ATCC 25922, *Pseudomonas aeruginosa* ATCC 27853. We solubilize the compounds **1**–**9** in DMSO at a concentration of 10 mg/mL. To determine the MICs of all the compounds, we prepared a serial twofold dilution of the tested compounds in Mueller-Hinton broth. The range of concentrations tested was: 5–0.019 mg/mL. Bacterial suspensions were prepared in sterile 0.9% saline, equivalent to the turbidity of a 0.5 McFarland standard, from 18–24 h microbial cultures obtained on TSB agar plates. We inoculate the 96 well microplates containing Mueller-Hinton broth with a twofold concentration of compounds solution with bacterial suspensions to a final density of 105 CFU/ mL per wells. After incubation at 37 °C for 18–24 h, the lowest concentrations of the compounds that inhibited visible bacterial growth were recorded as MICs. We determined the minimum bactericidal concentrations (MBCs) values by sub culturing wells on PCA (plate count agar) plates and incubated them at 37 °C for 18–24 h. After the incubation, the lowest concentration that did not show any visible growth was registered as the MBC.

### 2.7. Screening of the Antibiofilm Activity

The four bacterial reference strains were used to screen the potential anti-biofilm activity of the compounds, as previously reported [24]. Briefly, we discarded the planktonic bacteria from the wells of the microplate, then washed the wells three times with phosphate buffered saline (PBS), fixed them with cold methanol, and quantified their total biomass using the crystal violet assay. The intensity of the colored suspensions obtained after the addition of acetic acid 33% was measured spectrophotometrically at 492 nm. The lowest concentrations of compounds that inhibited biofilm formation on the plastic wells was recorded as the minimum inhibitory concentration of the biofilm development and we expressed the concentrations in mg/mL.

## 3. Results and Discussion

### 3.1. Synthesis and Structural Analysis

One can easily obtain the diphenylamino-derivatives employed in this study in a single- or two-steps, starting from accessible compounds. As we obtained compounds **2**–**4**, and **8**, using the methods already published in the literature, for these compounds, we only recorded the ^1^H- and ^13^C-NMR spectra to confirm their structure and to check their purity (see Supplementary Materials for details).

**Scheme 1:**
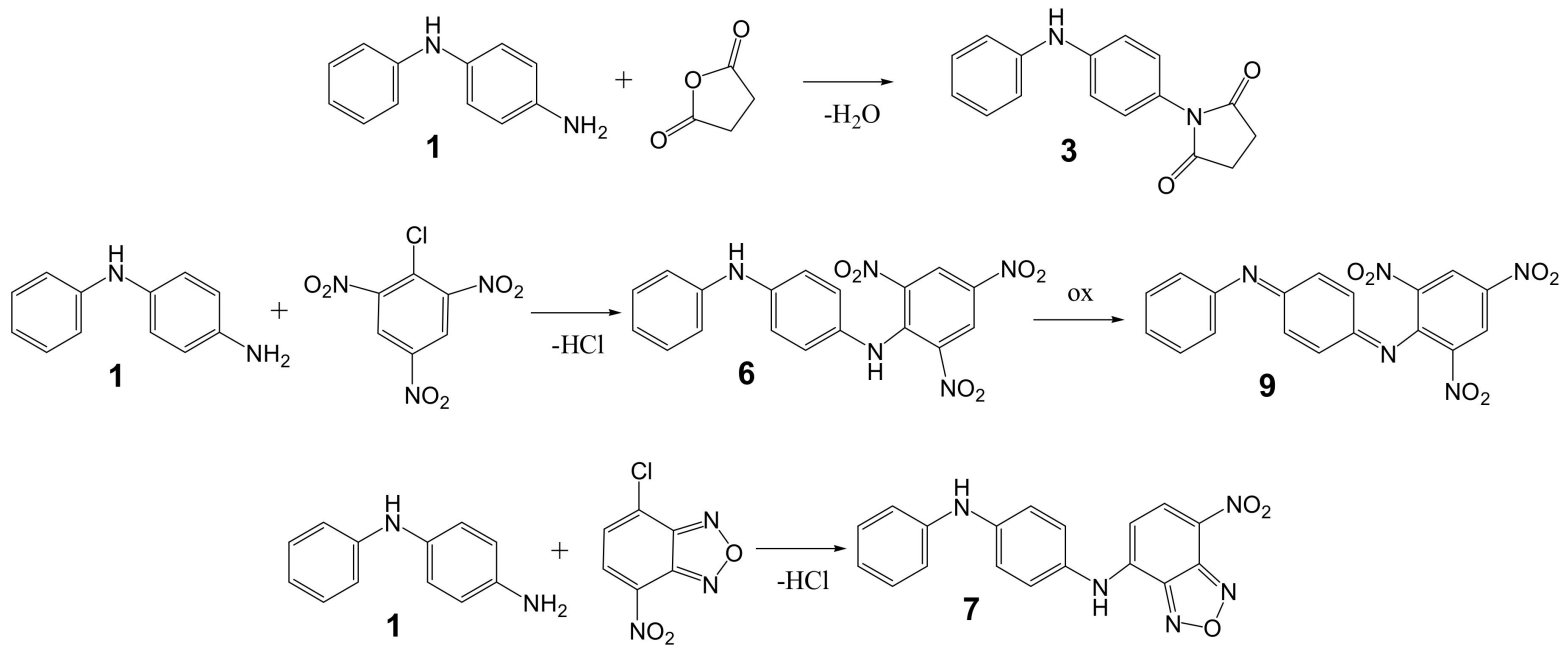
General procedure for the synthesis of the compounds.

Compounds **7** and **9** are novel synthesized derivatives of aminodiphenylamine (Scheme 1), and for this reason, we performed a full structural analysis (elemental analysis, IR, UV-Vis, ^1^H- and ^13^C-NMR, ESI-MS, X-ray diffraction on single crystal, where appropriate), confirming their structure and showing interesting features, as is presented next.

Firstly, the IR spectrum showed peaks related to the amine groups at 3307 cm^−1^, aromatic rings at about 2923 cm^−1^, and a nitro group at 1276 cm^−1^ (see Supplementary Materials, Figure S1). The UV-Vis spectrum showed the presence of an intense band at 497 nm, due to NBD moiety (Supplementary Materials, Figure S2). This compound did not show fluorescence, although many NBD derivatives are known for this behavior [25].

We noticed important details in the ^1^H-NMR spectrum. The two hydrogen nuclei from the NH groups appeared as singlets at 10.64 ppm and 7.78 ppm (see Experimental, Supplementary Materials, Figure S3, and Table 2). The two hydrogen nuclei from the NBD moiety appeared as a doublet, at 8.35 ppm and 6.53 ppm, whereas the other aromatic hydrogen nuclei were in the 7.22–8.81 ppm interval. The ^13^C-NMR spectrum was also consistent with the structure (Supplementary Materials, Figure S4). The ESI-MS spectrum recorded in negative mode showed the molecular peak at 346 *m*/*z*, again confirming the molecular structure (Supplementary Materials, Figure S5).

**Figure 2:**
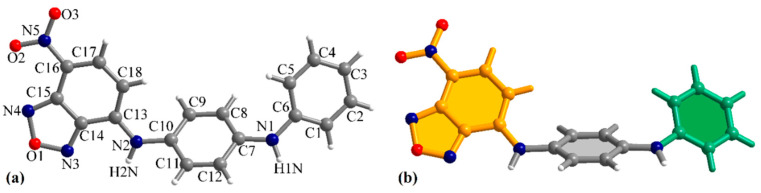
(**a**) View of the asymmetric unit in the crystal structure of compound **1** and (**b**) schematic representation of the planar moieties.

The solid-state structure of compound **7** was determined by X-ray diffraction on a single crystal. Compound **7** crystallized in the monoclinic P2_1_/c space group with one molecule in the asymmetric unit (Figure 2a). The structural characterization showed the dissymmetrical *N*,*N*’-disubstituted *p*-phenylenediamine derivative with nitro-benzofurazan and phenyl moieties. The nitro group was practically coplanar with the benzofurazan fragment. The dihedral angles between the mean planes of the *p*-phenylenediamine core and the nitro-benzofurazan, respectively phenyl fragments, are 44.0 and 43.6° (Figure 2b). In the nitro-benzofurazan fragment, the N-O bond lengths were: N3-O1 = 1.3635(16); N4-O1 = 1.3966(17); N5-O2 = 1.2298(17) and N5-O3 = 1.2401(18) Å. The C-N bond lengths are: C14-N3 = 1.3103(19); C15-N4 = 1.3160(19); C16-N5 = 1.4184(18); C6-N1 = 1.396(2); C7-N1 = 1.3928(18); C10-N2 = 1.4260(18) and C13-N2 = 1.3423(18) Å.

**Figure 3:**
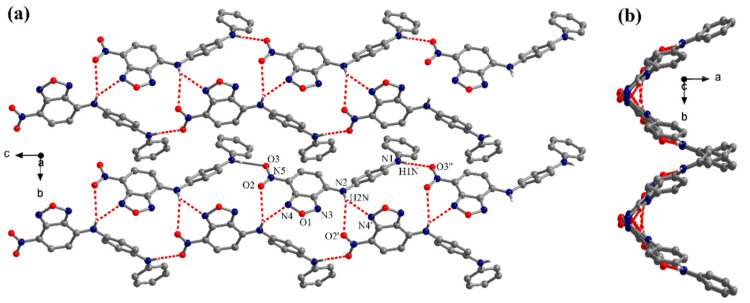
Views of the packing diagrams along the crystallographic *a* (**a**) and *c* axes (**b**).

The secondary amino groups were involved in hydrogen interactions with the nitro groups and oxadiazole rings belonging to neighboring molecules that generate supramolecular double chains running along the crystallographic *c* axis (Figure 3). The H1N atom interacted with the O3″ atom of a nitro group, whereas the H2N atom was involved in bifurcated interactions with the O2′ (nitro) and N4′ (oxadiazole) atoms (symmetry codes: ′ = x, 1.5 − y, −0.5 + z and ″ = x, y, −1 + z). The distances for the hydrogen interactions were: (N1)H1N··· O3″ = 2.202; (N2)H2N··· O2′ = 2.785 and (N2)H2N··· N4′ = 2.513 Å. The corresponding angles were: N1-H1N··· O3″ = 176.1; N2-H2N··· O2′ = 128.8 and N2-H2N··· N4′ = 144.2°.

### 3.2. Lipophilicity and Related Measurements

One of the best lipophilicity indicators (denoted as *R_M_*_0_) and the specific hydrophobic surface area (denoted as *b*) were taken into consideration [26,27] for compounds **1**–**9**, and these values are compiled in Table 2. The value of the specific hydrophobic surface area b represents a linear correlation that existed between the *R_M_* values of the compounds and the concentration (*C*) of the organic solvent (in our case acetone) in the eluent (a mixture of acetone and water). In this way, the intercept *R_M_*_0_ is practically the *R_M_* value of the compound that corresponds to the extrapolated zero organic phase concentration in the eluent. In the same manner, the slope *b* is the change in the lipophilicity caused by a unit concentration change in the organic phase [28,29].

The highest experimental *R_M_*_0_ values (Table 2) was recorded for compounds **6**, **7**, and **9**, whereas the lowest values were for recorded for compounds **1**–**3**. If the molecule contained supplementary hydrophobic moieties, such as aromatic rings, we expected the lipophilicity to be higher, and this was the trend that we noticed for our compounds. Inversely, polar groups reduce the lipophilicity, as noticed for compounds **1**–**3**. In a similar manner, we recorded the lowest specific hydrophobic surface *b* (Table 2) for compounds **6**, **7**, and **9**, whereas we recorded the highest for compounds **1**–**3.** The *R_f_* measurements values are also presented in Supplementary Materials, Table S1.

### 3.3. Total Antioxidant Capacity

The *TAC* values were obtained using the most-known DPPH method [30], based on the fading of the intense violet color of the free radical solution. We measured these values using Equation (3), where Abs_0_ refers to the initial absorbance of the DPPH free radical (at 517 nm) and *Abs*_30min_ refers to the absorbance recorded at the same wavelength after 30 min.

(3) $$TAC=\frac{Abs_{0}-Abs_{30\min}}{Abs_{0}}\times 100$$

From Table 2, one can see that compound **1** had the highest value (87%), which was an expected result, as amines are known for their easiness of oxidation. Compounds **3** and **5** also showed higher values, over 80%. For compounds **4**, **8**, and **9**, recording accurate values was not possible, either due to their low solubility in methanol or their high intense color which might have interfered with the DPPH one. Although these values should be considered only by comparing them with others, the information gained provides insights regarding the antioxidant potential of the derivatives employed in this study. The mechanism of action probably involves the well-known capacity of DPPH free radical to abstract one hydrogen atom from amine derivatives.

In order to gain supplementary information, we used also the ABTS method. This presents the possibility to monitor the reaction at different wavelengths [20,30] (the same Equation (3) was used to calculate *TAC*). An in the previous DPPH case, the highest value was recorded for the compound **1**, followed by **3**, but in this case, it was possible to obtain the TAC values for compounds **4**, **8**, and **9**, due to the different wavelength of the ABTS absorbance. 

### 3.4. Correlations between Experimental and Computed Properties

The correlation between the structure of the compound and its biological activity plays an important role in the study of the interaction between chemicals and living organisms. Thus, scholars widely employ QSAR techniques in such evaluations. Lipophilicity is one of the most important molecular properties, as the capacity of chemical compounds to cross the lipophile cell membrane is correlated with their biological activity in QSAR studies. Usually, scholars calculate the *log P* values by using the fragment method and including additions of the fragmental constants, based on the relationship $\log P=\sum_{1}^{n}a_{n}f_{n}$, where a stands for the occurrences of the type n structure for fragment *f*.

**Figure 4:**
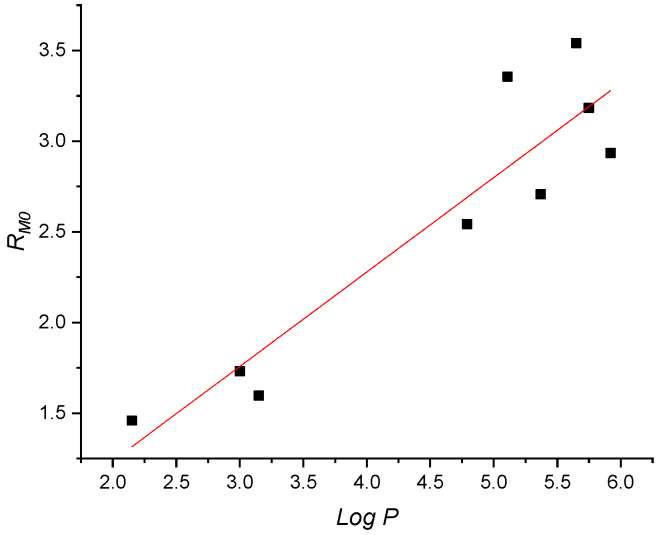
Correlation between experimental (*R_M_*_0_) and computed lipophilicity (*log P*).

To check if any correlation between the lipophilicity index experimentally measured (*R_M_*_0_) and the partition coefficient evaluated by the computational method (*log P*) exists, we plotted the values obtained first against the second ones (Figure 4). Although some scattering of the data points was present in Figure 4, the experimental values correlated well with the theoretical ones (*R* = 0.86). However, a very large number of derivatives is required for an accurate statistical assessment.

In addition to the classical correlation between the experimental *R_M_*_0_ lipophilicity and the computed one *log P*, the polar surface area (*PSA*) may provide some more information, as this parameter represents the sum of polar atoms surfaces contained by a specific molecule, and therefore is an indicator of hydrophobicity (as opposed to lipophilicity) [31,32]. Table 2 shows these values, and one can see that compound **6** had the highest *PSA* value recorded (161.53 A^2^) and the lowest specific hydrophobic surface *b* (−4.4791), demonstrating the intercorrelation between the lipophilic and hydrophobic balance.

To obtain a more enhanced understanding of the relationship between the chemical structure and properties, we employed some other computational measurements, such are atomic charges; as experimental data included the maximum absorption wavelength (λ_max_) of the compounds **1**–**9** and some of their δ NMR data (Table 2 shows selected values on nitrogen atoms for *Huckel AC* and δ for amine hydrogen). We choose this approach because these data (an NMR of amines and Huckel electron densities) correlate well [33]. Scholars have also developed methods to assess some structure–property relationships by computational study, using a Hückel approach [34]. Thus, physical measurements including UV/Vis spectra represent a simple and fast way to obtain structural information. Moreover, the use of molecular orbital theory has a long history and a certain future [35]. Regarding compounds **1**–**9**, as expected, we recorded the highest wavelengths (λ_max_) for the derivatives **6**–**9** (due to extended conjugation). Regarding the NMR values, the influence of the adjacent (poly)nitro-aromatic moiety on the -N*H*- group is reflected in the higher δ values recorded for compounds **6** and **7** (10.29 and 10.64 ppm, respectively). In a similar manner, we noticed these influences on the *N*-atomic charges (*Huckel AC*, Table 2).

### 3.5. Bioevaluation of the Compounds as Antimicrobial and Antibiofilm Agents

**Figure 5:**
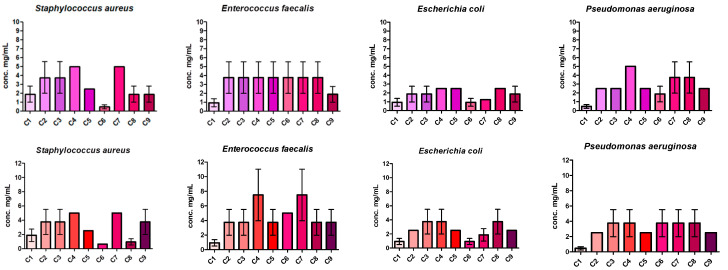
Top: MIC values obtained for compounds **1–9** for *S. aureus*, *E. faecalis*, *P. aeruginosa*, and *E. coli*; bottom: similarly, MBC values.

To investigate the compound antibacterial activity, we determined MIC and MBC values against four types of bacterial strains: *Staphylococcus aureus* ATCC 25923, *Enterococcus faecalis* ATCC 29212, *Escherichia coli* ATCC 25922, and *Pseudomonas aeruginosa* ATCC 27853. We found that compounds **1** and **6** were the most active, with MIC and MBC values in the range of 1.25–0.312 mg/mL (Figure 5). Compounds **2**, **3**, **4**, **5**, **7**, **8**, and **9** had MICs and MBCs between 1.25 and 5 mg/mL.

**Figure 6:**
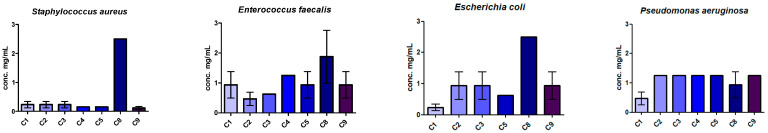
Antibiofilm activity of the employed compounds.

Bacteria are less susceptible to antibiotics when they are grown in biofilms. To determine if the compounds had antibiofilm proprieties, we also determined their effects on bacterial adherence. Our results showed that compound **1** was the most active in inhibiting the biofilm formation of all the bacterial strains tested, with MBICs values between 0.156 and 0.625 mg/mL. Compound **9** displayed the most pronounced antibiofilm activity, with MBIC of 0.078 mg/mL, against *S. aureus* ATCC 25923 (Figure 6). Compounds **2**–**9** were less active against the biofilm of *Enterococcus faecalis* ATCC 29212, *Escherichia coli* ATCC 25922, and *Pseudomonas aeruginosa* ATCC 27853, and the MBICs values ranged from 0.625 to 1.25 mg/mL.

## 4. Conclusions

Nine compounds were employed for lipophilicity and antioxidant studies, and we found that the structural characteristics of these compounds were correlated with some of their properties. New compounds derived from 4-aminodiphenylamine were synthesized and characterized by IR, NMR, UV-Vis, MS, etc. Additionally, we obtained suitable crystals for X-ray diffraction, and therefore we revealed the crystal structure for the compound **7**. The results of the antioxidant evaluation showed that the starting materials had higher TAC values. We also found a high correlation between the experimental *R_M_*_0_ values and calculated *log P*. In this way, RP-TLC can be regarded as a cheap and fast experimental alternative [36,37,38,39] for evaluating some lipophilicity descriptors. In addition, these compounds showed antimicrobial and antibiofilm activity.

## Figures and Tables

**Table 1:** Crystallographic data, details of data collection, and structure refinement parameters for compound **7**.

Compound	**7**
Chemical formula	C_18_H_13_N_5_O_3_
M (g mol^−1^)	347.33
Temperature, (K)	293 (2)
Wavelength, (Å)	0.71073
Crystal system	*Monoclinic*
Space group	*P*2_1_*/c*
*a* (Å)	13.0162(8)
*b* (Å)	9.6307 (5)
*c* (Å)	12.7982 (7)
*α* (°)	90
*β* (°)	93.333 (5)
γ (°)	90
V (Å^3^)	1601.61 (16)
Z	4
*D_c_* (g cm^−3^)	1.440
*μ* (mm^−1^)	0.103
*F*(000)	720
Goodness-of-fit on *F*^2^	1.058
Final *R*_1_, w*R*_2_ [I > 2σ(I)]	0.0363, 0.0958
*R*_1_, w*R*_2_ (all data)	0.0439, 0.1001

**Table 2:** Experimental and computed values of the compounds **1**–**9**.

Cmp.	λ_max_ (nm)	NMR ^a^ (*δ* ppm)	*Huckel AC* ^c^	*log P* ^d^	*PSA* ^d^ (A^2^)	*R_M_*_0_	*b*	*r* ^2^	*TAC* ^f^ (%)	*TAC* ^g^ (%)
**1**	285	5.39/3.51 ^b^	0.327/0.062	3.00	38.05	1.732	−2.5949	0.96	87	75
**2**	297	5.36/7.25	0.347/0.312	3.15	41.12	1.597	−2.8039	0.94	43	90
**3**	291	5.96/-	0.351/0.534	2.15	49.41	1.459	−2.4863	0.97	84	77
**4**	293	8.04/-	0.351/0.495	4.79	51.1	2.543	−3.4316	0.99	- ^e^	42
**5**	301	7.88/7.88 ^b^	0.338/0.338	5.92	24.05	2.935	−3.7514	0.99	81	61
**6**	422	5.82/10.29	0.383/0.485	5.65	161.53	3.541	−4.4791	0.97	60	66
**7**	497	7.78/10.64	0.377/0.518	5.75	108.8	3.184	−4.1715	0.97	47	- ^e^
**8**	440	-/-	0.158/0.158	5.37	24.73	2.707	−3.4197	0.98	- ^e^	15
**9**	400	-/-	0.596/−0.308	5.11	162.2	3.356	−4.1594	0.97	- ^e^	14

^a^ values recorded for the H-nucleus of NH-group(s); ^b^ literature data; ^c^ calculated using ChemBio3D for hydrazine nitrogen atoms; ^d^ calculated using MolInspiration; ^e^ low solubility of the compound in methanol and/or absorbance around 517 or 730 nm; ^f^ DPPH method; ^g^ ABTS method.

## Data Availability

The data is contained within the article and Supplementary Materials.

## Supplementary Materials

The following supporting information can be downloaded at: https://www.mdpi.com/article/10.3390/antiox12030713/s1, Figure S1. IR spectrum of **7**; Figure S2. UV-Vis spectrum of **7**; Figure S3. ^13^C-NMR spectrum of **7**; Figure S4. -ESI-MS spectrum of **7**; Table S1; *R_f_* values of compounds **1**–**9** at different concentration acetone/water (*C*) mixtures on RP-TLC plates (C18, F254); synthetic details and structural analysis for compounds **2**–**4**, **6** and **8**.
