# Supplementary material for: Biological Evaluation and Structural Analysis of Some Aminodiphenylamine Derivatives
Source: Antioxidants (Basel). 2023 Mar 13;12(3):713. doi: 10.3390/antiox12030713 (PMC10045258; doi:10.3390/antiox12030713)
Supplement: Supplementary file 1 [file antioxidants-12-00713-s001.zip › antioxidants-2229308-supplementary.pdf]

Supplementary Materials

Biological Evaluation and Structural Analysis of Some Aminodiphenylamine Derivatives

A. Bujor, A. Hanganu, V. Tecuceanu, A. M. Madalan, M. Tudose, L. Marutescu, M. Popa, C. M. Chifiriuc, I. Zarafu, P. Ionita

---

Figure S1. IR spectrum of **7.**

Figure S2. UV-Vis spectrum of **7.**

Figure S3. ^{1}H-NMR spectrum of **7.**

Figure S4. ^{13}C-NMR spectrum of **7.**

Figure S5. -ESI-MS spectrum of **7.**

Table S1. R_f values of compounds **1-9** at different concentration acetone/water (C) mixtures on RP-TLC plates (C18, F254).

Synthetic details and structural analysis for compounds 2-4, 6 and 8.

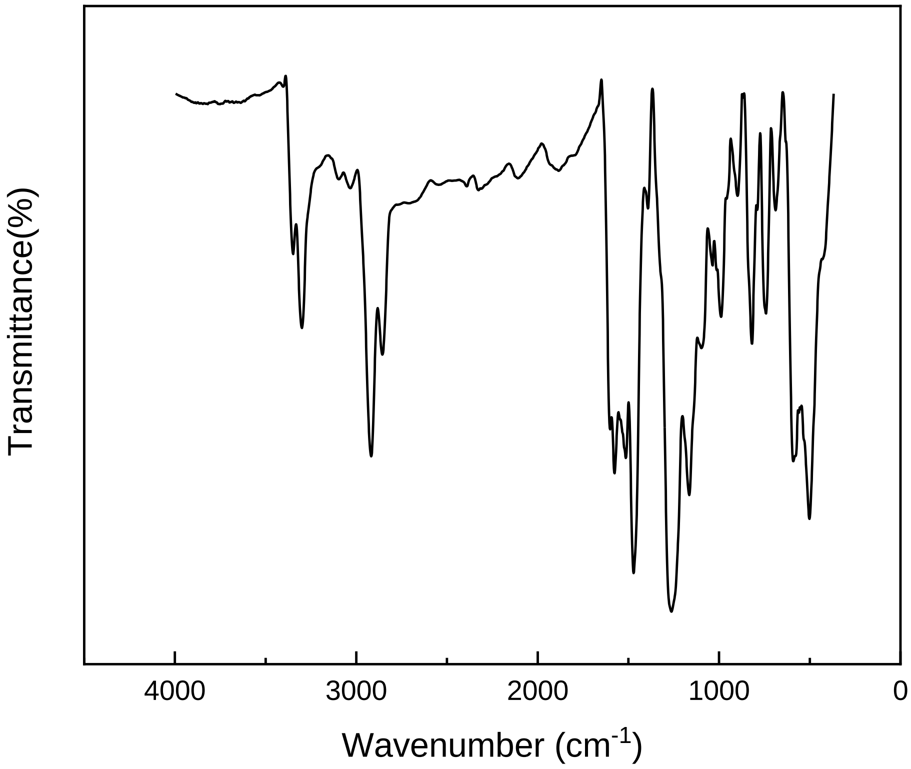

Figure S1. IR spectrum of **7.**

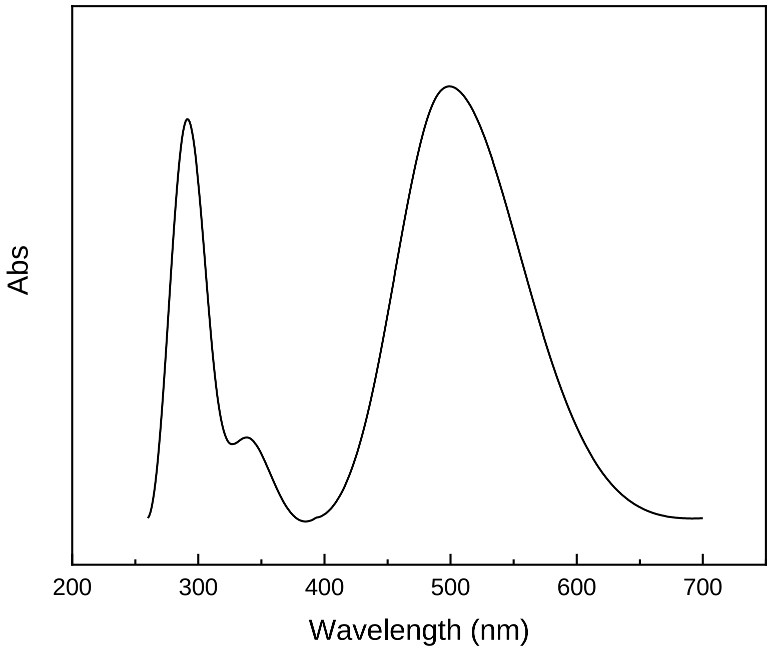

Figure S2. UV-Vis spectrum of **7.**

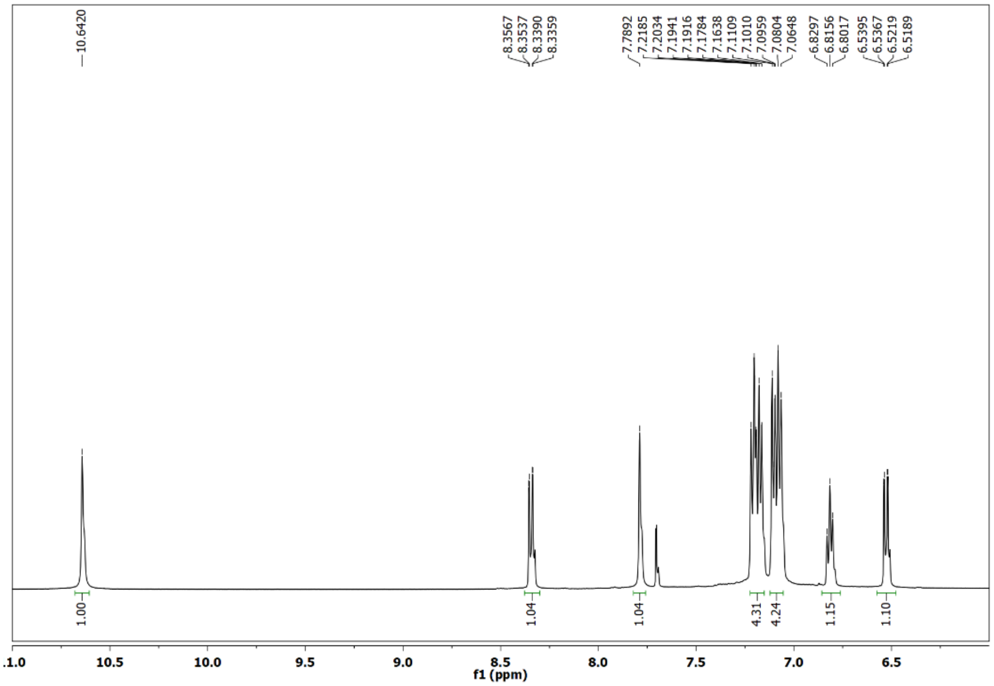

Figure S3. ^{1}H-NMR spectrum of **7.**

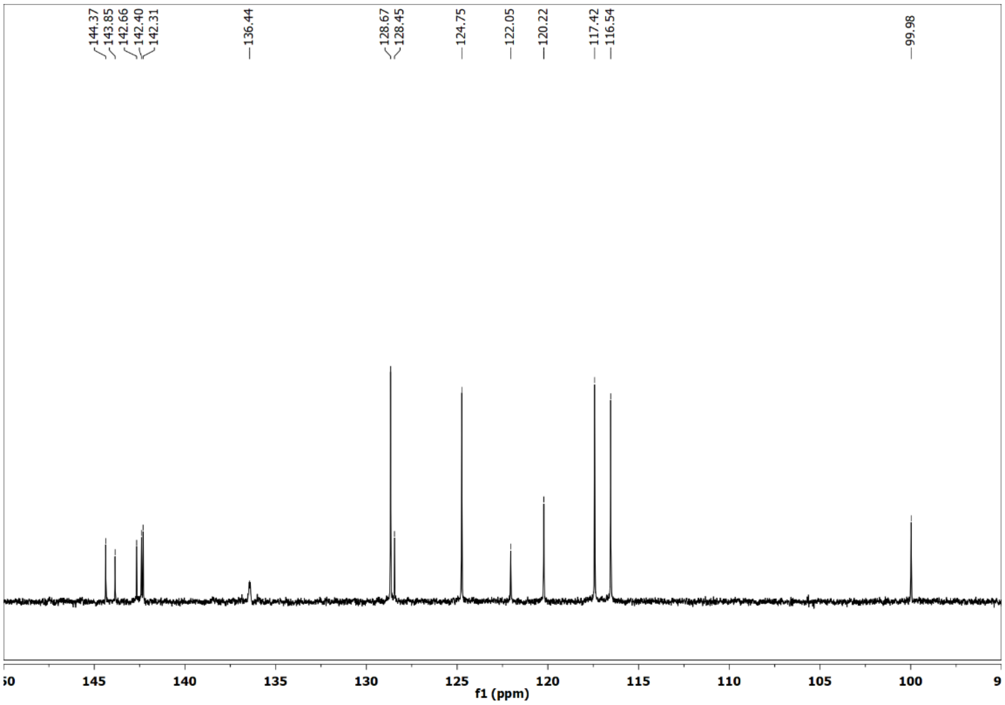

Figure S4. ^{13}C-NMR spectrum of **7.**

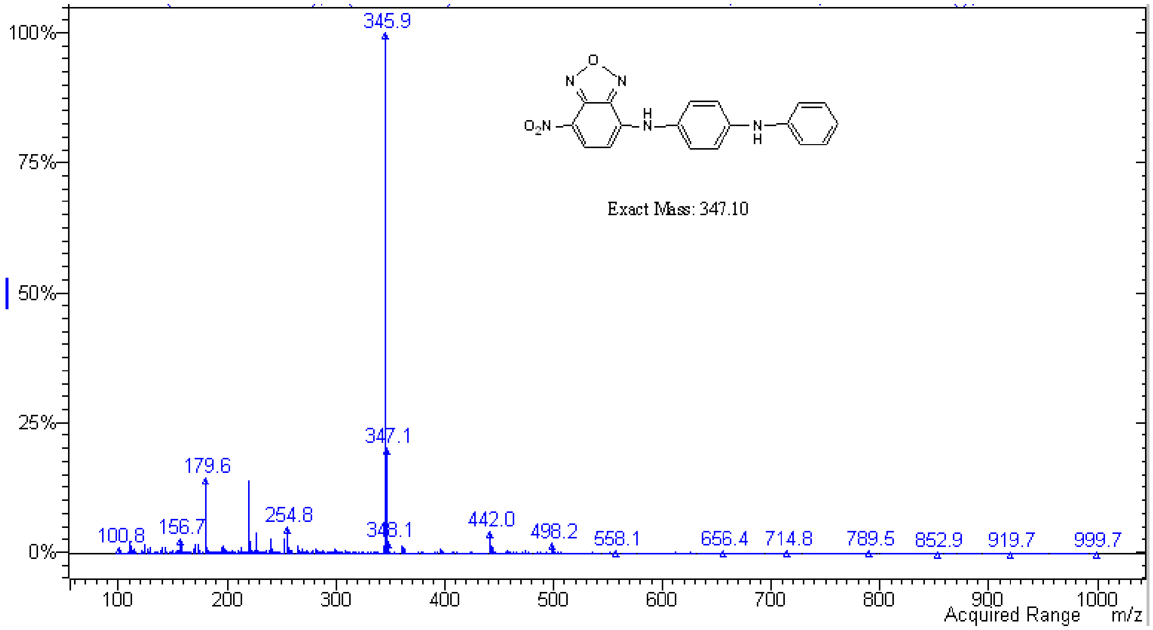

Figure S5. -ESI-MS spectrum of **7.**

Table S1. R_f values of compounds **1-9** at different concentration acetone/water (*C*) mixtures on RP-TLC plates (C18, F254).

	$\boldsymbol{R_f}$			
Cmp./*C*	0.6	0.7	0.8	0.9
**1**	0.41	0.56	0.64	0.82
**2**	0.57	0.7	0.77	0.91
**3**	0.53	0.66	0.74	0.87
**4**	0.24	0.44	0.6	0.78
**5**	0.17	0.34	0.52	0.74
**6**	0.11	0.34	0.48	0.76
**7**	0.15	0.42	0.57	0.78
**8**	0.17	0.36	0.5	0.7
**9**	0.11	0.32	0.43	0.72

Synthetic details and structural analysis for compounds 2-4, 6 and 8.

Compound **2**, *4-(acetylamino)diphenylamine*. To 1.84 g (0.01 mol) of compound **1** dissolved in 30 mL acetic acid was added 1.02 g acetic anhydride (0.01 mol) and the mixture was stirred for 24 h, followed by reflux for 1 min. After cooling, the addition of ice-cold water led to precipitation of the derivative. Yield 88%. ^{1}H-NMR (500 MHz, $CDCl_3$, δ ppm, J Hz): 7.37 (d, 2H, H_{Ar}, J = 8.5 Hz), 7.28-7.25 (m, 2H, H_{Ar}, overlapped with $CDCl_3$ signal), 7.09-6.96 (m, 5H, H_{Ar}), 2.16 (s, 3H, CH_3) ppm. ^{13}C-NMR (125 MHz, $CDCl_3$, δ ppm): 168.26, 148.09, 143.51, 139.66, 129.46, 127.73, 121.69, 118.87, 117.53, 24.36 ppm.

Compound **3**, *1-(4-anilinophenyl)pyrrolidine-2,5-dione.* After the reflux for 4 h of a mixture of 1.84 g (0.01 mol) of **1** and 1 g (0.01 mol) of succinic anhydride in 100 mL of acetic acid, the addition of ice-cold water led to the precipitation of the desired compound. Yield 75%. ^{1}H-NMR (500 MHz, $CDCl_3$, δ ppm, J Hz): 7.28 (t, 2H, H_{Ar}, J = 7.7 Hz), 7.10-7.07 (m, 6H, H_{Ar}), 6.98 (t, 1H, H_{Ar}, J = 7.3 Hz), 5.96 (s, 1H, N*H*), 2.85 (s, 4H, CH_2) ppm. ^{13}C-NMR (125 MHz, $CDCl_3$, δ ppm): 176.62, 143.80, 142.04, 129.33, 127.42, 123.75, 121.79, 118.78, 116.95, 28.29

Compound **4**, *N-(phenylaminophenyl)phtalimide.* After the reflux for 4 h of a mixture of 1.84 g (0.01 mol) of **1** and 1.48 g (0.01 mol) of phthalic anhydride in 100 mL of acetic acid, the addition of ice-cold water led to the precipitation of the desired compound. Yield 95%. ^{1}H-NMR (500 MHz, DMSO-d_6, δ ppm, J Hz): 8.04 (s, 1H, N*H*), 7.95-7.93 (m, 2H, H_{Ar}), 7.90-7.88 (m, 2H, H_{Ar}), 7.29-7.25 (m, 4H, H_{Ar}), 7.18-7.14 (m, 4H, H_{Ar}), 6.88 (t, 1H, H_{Ar}, J = 7.3 Hz) ppm. ^{13}C-NMR (125 MHz, DMSO-d_6, δ ppm): 167.39, 143.53, 142.79, 134.59, 131.58, 129.26, 128.42, 123.30, 123.00, 120.35, 117.42, 116.02.

Compound **6**, *N-picryl-4-aminodiphenylamine.* To 0.9 g (5 mmol) of **1** dissolved in 30 mL methanol was added 1.3 g (5.3 mmol) of picryl chloride and 3 g of sodium hydrogen-carbonate and the mixture warmed slowly. Next day addition of diluted hydrochloric acid led to the precipitation of the desired compound; alternatively, can be extracted with DCM. Yield 83%. ^{1}H-NMR (500 MHz, $CDCl_3$, δ ppm, J Hz): 10.29 (s, 1H, N*H*), 9.05 (s, 2H, H_{Ar}), 7.31 (t, 2H, H_{Ar}, J = 7.8 Hz), 7.09 (d, 2H, H_{Ar}, J = 8.2 Hz), 7.02-6.95 (m, 5H, H_{Ar}), 5.82 (s, 1H, N*H*) ppm. ^{13}C-NMR (125 MHz, $CDCl_3$, δ ppm): 143.32, 141.60, 139.48, 137.54, 134.92, 129.53, 129.51, 127.30, 122.90, 122.36, 119.18, 117.30.

Compound **8**, *diphenyl-p-phenylenediimine.* 0.5 g (2 mmol) of **5** dissolved in 50 mL DCM was stirred for 24 h with 5 g of lead dioxide. Filtration and removal of the solvent afforded the desired compound. Yield 65%. ^{1}H-NMR (500 MHz, $CDCl_3$, δ ppm, J Hz): 7.41-7.34 (m, 4H, H_{Ar}), 7.20-7.15 (m, 2H, H_{Ar}), 7.11 (d, 1H, H_{Ar}, J = 1.1 Hz), 6.97 (dd, 1H, H_{Ar}, J = 1.9 Hz, J = 10.2 Hz), 6.92-6.86 (m, 5H, H_{Ar}), 6.74 (d, 1H, H_{Ar}, J = 1.1 Hz) ppm. ^{13}C-NMR (125 MHz, $CDCl_3$, δ ppm): 158.47, 158.38, 150.01, 149.99, 137.71, 136.54, 128.99, 128.93, 125.21, 125.16, 120.61, 120.54.
